# *ETV6*/*RUNX1* Fusion Gene Abrogation Decreases the Oncogenicity of Tumour Cells in a Preclinical Model of Acute Lymphoblastic Leukaemia

**DOI:** 10.3390/cells9010215

**Published:** 2020-01-15

**Authors:** Adrián Montaño, Jose Luis Ordoñez, Verónica Alonso-Pérez, Jesús Hernández-Sánchez, Sandra Santos, Teresa González, Rocío Benito, Ignacio García-Tuñón, Jesús María Hernández-Rivas

**Affiliations:** 1 IBSAL, IBMCC, Cancer Research Center, Universidad de Salamanca-CSIC, 37007 Salamanca, Spain; adrianmo18@gmail.com (A.M.); jlog@usal.es (J.L.O.); alonsoperezveronica@gmail.com (V.A.-P.); jesus807@gmail.com (J.H.-S.); sandruskism90@gmail.com (S.S.); teresa.gonzalez@mundo-r.com (T.G.); beniroc@usal.es (R.B.); 2 Department of Biochemistry and Molecular Biology, University of Salamanca, Campus Unamuno s/n, 37007 Salamanca, Spain; 3 Department of Hematology, Hospital Universitario de Salamanca, 37007 Salamanca, Spain; 4 Department of Medicine, Universidad de Salamanca and CIBERONC, 37007 Salamanca, Spain

**Keywords:** acute lymphoblastic leukaemia, ETV6/RUNX1, CRISPR/Cas9, genome edition

## Abstract

Background: The t(12;21)(p13;q22), which fuses *ETV6* and *RUNX1* genes, is the most common genetic abnormality in children with B-cell precursor acute lymphoblastic leukaemia. The implication of the fusion protein in leukemogenesis seems to be clear. However, its role in the maintenance of the disease continues to be controversial. Methods: Generation of an in vitro *ETV6*/*RUNX1* knock out model using the CRISPR/Cas9 gene editing system. Functional characterization by RNA sequencing, proliferation assays, apoptosis and pharmacologic studies, and generation of edited-cell xenograft model. Results: The expression of *ETV6*/*RUNX1* fusion gene was completely eliminated, thus generating a powerful model on which to study the role of the fusion gene in leukemic cells. The loss of fusion gene expression led to the deregulation of biological processes affecting survival such as apoptosis resistance and cell proliferation capacity. Tumour cells showed higher levels of apoptosis, lower proliferation rate and a greater sensitivity to PI3K inhibitors in vitro along as a decrease in tumour growth in xenografts models after *ETV6*/*RUNX1* fusion gene abrogation. Conclusions: ETV6/RUNX1 fusion protein seems to play an important role in the maintenance of the leukemic phenotype and could thus become a potential therapeutic target.

## 1. Background

The gene fusion between the transcription factors *ETV6* (*TEL*) and *RUNX1* (*AML1*), generated by t(12;21)(p13;q22), is the most frequent chromosomal translocation in children with acute lymphoblastic leukaemia (ALL). This translocation fuses the 5′ non-DNA binding region of the ETS family transcription factor *ETV6* to almost the entire *RUNX1* locus [1,2]. Patients carrying this translocation are associated with a good prognosis and excellent molecular response to treatment. However up to 20% of cases relapse [3,4,5,6,7]. Furthermore, the response to treatment of some relapse cases is associated with resistance to treatments such as glucocorticoids (GCs) [8], and these patients must be treated with stem cell transplantation [9].

ETV6/RUNX1 (E/R) protein is known to play a role in the development of B-ALL, but by itself it is not capable of initiating the disease. Postnatal genetic events are required for the development of clinically overt leukaemia. These second events are usually mutations or deletions, such as the loss of wild type (WT) allele of *ETV6* [10]. Recent studies suggest that E/R is responsible for the initiation of leukaemia and is also essential for disease progression and maintenance, through deregulation of different molecular pathways that contribute to leukemogenesis. E/R regulates phosphoinositide 3-kinase (PI3K)/Akt/mammalian target of rapamycin (mTOR) (PI3K/Akt/mTOR) pathway, which promotes proliferation, cell adhesion and DNA damage response; *STAT3* pathway involved in self-renewal and cell survival and *MDM2*/*TP53* whose deregulation induces the inhibition of apoptosis and consequently cell survival [11].

However, the functional studies carried out by the silencing of *E*/*R* fusion gene expression, mediated by siRNA and shRNA, reveal that there is still controversy about the role of the oncoprotein in the maintenance of the leukemic phenotype. Thus E/R silencing by siRNA neither induced cell cycle arrest/apoptosis nor attenuated clonogenic potential of cells. Therefore, the E/R fusion protein may be dispensable for the survival of definitive leukemic cells [12]. By contrast, other studies showed that E/R expression was critical for the survival and propagation of the respective leukaemia cells in vitro and in vivo [13,14]. These results arise some doubts about the implications of the fusion protein in tumour cells.

The implementation of new genetic editing strategies has allowed the development of functional studies by generation of gene and gene fusion Knock-out (KO) models, both in vitro and in vivo [15]. In this study, we completely abrogated the expression of E/R fusion protein in REH ALL cell line using the CRISPR/Cas9 editing system and we observed the deregulation of different biological processes such as apoptosis resistance and cell proliferation. Consequently, leukaemia cells showed greater sensitivity to death and less proliferative advantage after gene fusion abrogation. E/R KO cells also showed an increased sensitivity to PI3K inhibitors and a decrease of the oncogenicity in vivo. In summary, we provide evidence that fusion protein has a key role in the maintenance of the leukemic phenotype.

## 2. Material and Methods

### 2.1. Cell Lines and Culture Conditions

REH, obtained from Deutsche Sammlung von Mikroorganismen und Zellkulturen (DMSZ) German collection (ACC 22), is a cell line established from the peripheral blood of a patient with ALL who carried t (12,21) and del(12) producing respective *E*/*R* fusion and deletion of residual *ETV6*. REH was maintained in RPMI 1640 (Life Technologies, Carlsbad, CA, USA) supplemented with 15% foetal bovine serum (FBS) and 1% of Penicillin/Streptomycin (P/S) (Life Technologies). Stromal HS-5 cell line was obtained from American Type Culture Collection (ATCC) collection (CRL-11882) and maintained in DMEM (Life Technologies, Carlsbad, CA, USA) supplemented with 10% FBS and 1% of P/S. Both cells lines were maintained at 37 °C with 5% CO_2_.

### 2.2. Sgrnas Design and Cloning

Based on the methodology of CRISPR/Cas9, two single guides RNAs (sgRNAs) (G1 and G2) were designed with the Broad Institute CRISPR designs software (http://www.broadinstitute.org). One of them directed towards the end of exon 5 of *ETV6* and other directed towards the beginning of intron 5–6, both before the fusion point, with the intention of producing indels or deletions that modify the open reading frame of the oncogene, and, therefore, the gene expression. These sgRNAs were cloned into a vector containing the Cas9 nuclease coding sequence and GFP, pSpCas9(BB)-2A-GFP (PX458) (Addgene plasmid #48138) (Ran 2013) as described previously [15] (Table S1). Then, they were electroporated into the REH cells.

### 2.3. Sgrna Transfections

REH ALL cells (4 × 10^6^ cells) were electroporated with 4 µg of both plasmid constructs (Garcia tunon 2017) (PX458 G1 and PX458 G2) using the Amaxa electroporation system (Amaxa Biosystem, Gaithersburg, MD, USA) according to supplier’s protocol.

### 2.4. Flow Cytometry Analysis and Cell Sorting

Seventy-two hours after sgRNAs transfection, GFP-positive cells were selected by fluorescence-activated cell sorting (FACS) using FACS Aria (BD Biosciences, San Jose, CA, USA). Single-cells were seeded in 96-well plate by FACS, establishing the different KO and control clones.

### 2.5. Sequencing of sgrNA Targets Sites

Genomic DNA was extracted using the QIAamp DNA Micro Kit (Qiagen, Hilden, Germany) following the manufacturer’s protocol. To amplify the region of *E*/*R* fusion, PCR was performed using the following primers: forward 5′-ACCCTCTGATCCTGAACCCC–3′ and reverse 5′-GGATTTAGCCTCATCCAAGCAG–3′. PCR products were purified using a High Pure PCR Product Purification Kit (Roche, Basilea, Switzerland) and were sequenced by the Sanger method using each forward and reverse PCR primers (Table S2).

The editing efficiency of the sgRNAs and the potential induced mutations were assessed using Tracking of Indels by Decomposition (TIDE) software (https://tide-calculator.nki.nl; Netherlands Cancer Institute), which only required two Sanger sequencing runs from wild-type cells and mutated cells.

### 2.6. Off-Target Sequence Analysis

The top four predicted off-target sites obtained from “Breaking Cas” website (http://bioinfogp.cnb.csic.es/tools/breakingcas/) were analysed by PCR in the different clones (Table S2) before to functional and xenograft experiments.

### 2.7. RT-qPCR

Total RNA extraction was performed with the RNeasy Kit (Qiagen) as suggested by the manufacturer. Real-time reverse transcriptase–polymerase chain reactions (RT-qPCRs) were performed as described [16]. The primers for *E*/*R* (sense, 5-CTCTGTCTCCCCGCCTGAA-3 antisense, 5-CGGCTCGTGCTGGCAT-3), were designed. These oligonucleotides were designed outside the editing region (exon 5 of *ETV6* and exon 4 of *RUNX1*) and with a distance between them in mRNA of 143 base pairs (bps) (Figure S2). RT-qPCR data shown include at least 3 independent experiments with 3 replicates per experiment.

### 2.8. Transcriptome Sequencing

RNA-seq was performed by using TruSeq Stranded mRNA (Illumina). In all samples, RNA was analysed following manufacturer’s recommendations for the protocol “TruSeq Stranded mRNA Reference Guide-Illumina”. Libraries were sequenced in the NextSeq550 platform (Illumina) according to manufacturer’s description with a read length of 2 × 75 nucleotides.

Briefly, bcl files were demultiplexing on BaseSpace (Illumina Cloud based resource) to generate fastq files. Raw data quality control was performed with fastQc (v0.11.8), globin contamination was assessed with HTSeq Count, FastQ screen evaluated ribosomal RNA contamination and other external possible resources of contamination (*Mus musculus*, *Drosophila melanogaster*, *Caenorhabditis elegans* and *mycoplasma*). STAR (v020201) was used for the alignment (hg19 reference genome) and Feature Counts (v1.4.6, University of Melbourne, Parkville, Australia) to generate the read count matrix. Finally, DESeq2 was used for differentially gene expression analysis. DESeq2 model internally corrects for library size therefore normalizes the values and enables paired comparisons. Heatmap was performed in R.

Go enrichment analysis (http://geneontology.org) to evaluate whether a set of genes was significantly enriched between the different comparisons was used. The most significant biological mechanisms, pathways and functional categories in the data sets of genes selected by statistical analysis were identified through PANTHER Overrepresentation Test. REVIGO was used to cluster biological processes (http://revigo.irb.hr) [17].

The data discussed in this publication have been deposited in NCBI’s Gene Expression Omnibus (Hernandez-Rivas JM et al., 2019) and are accessible through GEO Series accession number GSE140980 (https://www.ncbi.nlm.nih.gov/geo/query/acc.cgi?acc=GSE140980).

### 2.9. Western Blotting

Protein expression was assessed by SDS-PAGE and western blotting (WB). The antibodies were obtained from Cell Signalling Technology (Danvers, MA, USA) including a human anti-Bcl-2 antibody (1:1000; 2872) for Bcl-2, a human anti-Bcl-xL antibody (1:1000; 2762) for Bcl-xL, a human anti-phospho Akt antibody (1:1000; 4060) for p-Akt (Ser473) and a human anti-phospho mTOR antibody (1:1000;2971) for p-mTOR (Ser2448). Anti-rabbit IgG horseradish peroxidase-conjugated (1:5000, 7074) was used as a secondary antibody. Antibodies were detected using ECL^TM^ WB Detection Reagents (RPN2209, GE Healthcare, Chicago, IL, USA). ImageJ software was used for densitometric analysis [18,19].

### 2.10. Cell Viability, Cell Cycle Analysis and Proliferation Assays

Cell viability was measured by flow cytometry based on non-vital dye propidium iodide (PI) labelling. Briefly, 5 × 10^5^ cells were collected and washed twice in PBS and labelled with PI, allowing the discrimination of living-intact cells (PI-negative) and apoptotic cells (PI-positive). In parallel, cell distribution in the cell cycle phase was also analysed by measuring DNA content (PI labelling after cell permeabilization). These experiments were carried out after 24, 48 and 72 culture hours.

For proliferation measuring, MTT assays and labelling of cells with CellTrace Carboxyfluorescein Succinimidyl Ester (CFSE) Cell Proliferation Kit (Thermo Fisher, Madison, WI, USA) were used. In MTT assays, cells were plated on 96-well plates, cell density varied according to the days of the experiment, in a range between 3 × 10^4^ and 5 × 10^3^ cells (24–240 h). MTT solution (3-(4,5-cimethylthiazol-2-yl)-2,5-diphenyl tetrazolium bromide) was added at concentration of 0.5 μg/μL (Merck, Darmstadt, Germany). After incubation for 3–4 h at 37 °C, cells were lysed with the solubilization solution (10% SDS in 0.01M HCl) and absorbance was measured in a plate reader at 570 nm. For labelling of cells, 3 × 10^5^ cells were stained with CellTrace-CFSE following the manufacturer’s instructions and plated on 6-well plates. After 48 h, CFSE expression was measured by flow cytometry.

### 2.11. B-ALL-Stromal Cell Co-Culture

HS-5 human mesenchymal stromal cells (MSCs) were plated at a density of 1 × 10^5^ cells per coverslip in 6-well plates. After 24 h, control cells and E/R KO clones were stained with Celltrace-CFSE and 3 × 10^5^ cells placed on top of the stromal cell monolayer. Cells were co-cultured during 48h in RPMI 1640 supplemented with 15% FBS and 1% of P/S at 37 °C with 5% CO_2_.

### 2.12. Drugs and Treatments

The followings drugs were used: Vincristine and Copanlisib (BAY 80–6946) were obtained from Selleckchem (Houston, TX, USA) and Prednisolone (P6004) obtained from Merck. All drugs were prepared at the appropriate stocking concentrations in DMSO (Merck) and stored at −20 °C until use.

### 2.13. Mouse Xenograft Tumourigenesis

Sixteen four to five-week-old female NOD/SCID/IL2 receptor gamma chain null (NSG) mice (Charles River, Barcelona, Spain) were used. 5 × 10^6^ tumour cells from REH or control clone were subcutaneously injected into the left flank and tumour cells from KO clones (KO1, KO2 and KO3) were injected in the right flank as described previously [15]. Groups were established as follows: REH vs. KO2 in group 1, REH vs. KO3 in group 2, control clone vs. KO1 in group 3 and control clone vs. KO2 in group 4 (4 mice per group). The study received prior approval from the Bioethics Committee of our institution and followed the Spanish and European Union guidelines for animal experimentation (RD 53/2013 and 2010/63/UE).

Tumour diameters were measured every 2–3 days with a calliper. Tumour volume was calculated as described elsewhere by the formula a2bπ/6 (a and b being, respectively, the smallest and the biggest diameters). Mice were sacrificed by anaesthesia overdose when tumour volume reached 2 cm^3^ or 48–62 days after cell injection, upon which the tumours were collected and weighted.

### 2.14. Histopathology and Immunohistochemistry

Excised tumours were sampled just after sacrifice and representative areas were (a) formalin-fixed (24 h) (Merck Millipore, Burlington, MA, USA) and paraffin-embedded and (b) snap-frozen in OCT and stored at 80 °C as previously described [20]. Tissue sections 2 μM thick were stained with hematoxilin & eosin (H&E) and prepared for immunohistochemistry (IHC). IHC was performed as previously described [20] using the anti-Ki67primary antibody (Merck Millipore). The number of mitotic figures were counted in 6 high-power field.

### 2.15. Statistical Analysis

Statistical analysis was performed using GraphPad Prism 6 Software (San Diego, CA, USA). Differences in relative expression of E/R and cell viability after treatments were tested by Tukey’s multiple comparisons test. Differences in protein expression, proliferation and apoptosis levels were tested by unpaired *t*-test. Differences in tumour masses over time were tested by non-parametric Mann Whitney U test followed by Tukey’s multiple comparisons test and parametric Student’s t test. Statistical significance at values of *p* ≤ 0.05 (*), *p* ≤ 0.005 (**) and *p* ≤ 0.001 (***) was noted.

## 3. Results

### 3.1. CRISPR/Cas9 Edited Lymphoid Cell Line Showed a Loss of E/R Functionality

The fusion gene product between *ETV6* and *RUNX1* genes is generated by the union of *ETV6* intron 5 and *RUNX1* intron 1–2. *E*/*R* sequence was edited by CRISPR/Cas9 and evaluated by Sanger sequencing in REH cells (Figure S1). The edition efficiency evaluated through TIDE was 76.5% with sgRNA G1 and 86.2% with sgRNA G2. The most frequent generated mutations were insertions up to 4 bps.

Single-edited cells were seeded into a 96 well plate to obtain clones with a single edition that predicted a KO sequence for the oncogene. Only 48 single cell clones proliferated in culture. These clones were screened by sanger sequencing and the results revealed that more than 50% of clones (25/48) presented an edited *E*/*R* sequence. Among them, three single-edited cell clones “KO1, KO2 and KO3 clones” with a predicted *E*/*R* KO sequence were selected for the study. These clones had different editions in their sequences. KO1 clone carried an insertion of two cytosines at the end of *ETV6* exon 5, near the Protospacer Adjacent Motif (PAM) sequence of the sgRNA G2, thus a frameshift mutation that generated a stop codon before finishing the exon. On the other hand, KO2 and KO3 had an insertion of 5 and 3 nucleotides respectively near the PAM sequence within exon 5, followed by a deletion of 100 bps approximately between both sgRNAs. These alterations modified the open reading frame, generating the stop codon in the next exon. In addition, the loss of the splicing region prevented the correct processing of the protein. Additionally, two single-edited cell clones with WT *E*/*R* sequence were used as control clones “Control 1 and Control 2” (Figure S2).

**Figure 1:**
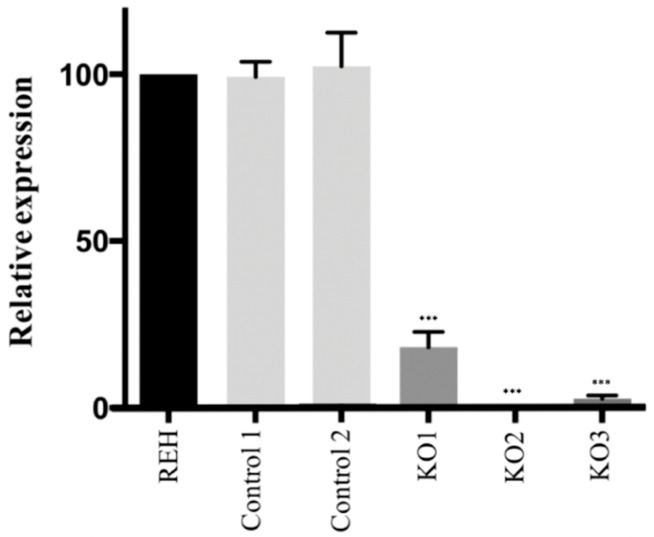
E/R expression levels by Reverse Transcription–quantitative real-time Polymerase Chain Reaction (RT-qPCR). Control clones showed an expression of E/R similar to it was observed in the parental REH cells. In E/R KO clones, whose sequence was edited by the CRISPR/Cas9 system, KO2 and KO3 showed a total loss of E/R expression and KO1 showed a leaky expression. All the experiments were carried out by triplicate, the means with the standard deviations for each clone were represented. *** *p* ≤ 0.001 (unpaired *t*-test).

In order to check the functionality of the *E*/*R* alleles carrying these clones, the expression of the fusion transcript E/R was quantified by RT-qPCR. Quantification revealed a total loss of E/R mRNA expression in KO2 and KO3 clones as compared to control clones (*p* < 0.001) and a leaky expression in KO1 clone (*p* < 0.001) (Figure 1). The loss of E/R expression was also observed by total RNA-seq in E/R KO clones.

The four most likely off-target sequences from both guides were analysed by Sanger sequencing into the different clones. Results of Sanger sequencing revealed the lack of editing in those regions, confirming the absence of CRISPR/Cas9 off-targets (data not shown).

### 3.2. Transcriptomic Analysis of E/R KO Lymphoid Cell Line Generated by CRISPR/Cas9 Showed a Distinct Expression Signature and a Deregulation of Its Downstream Signalling Genes

**Figure 2:**
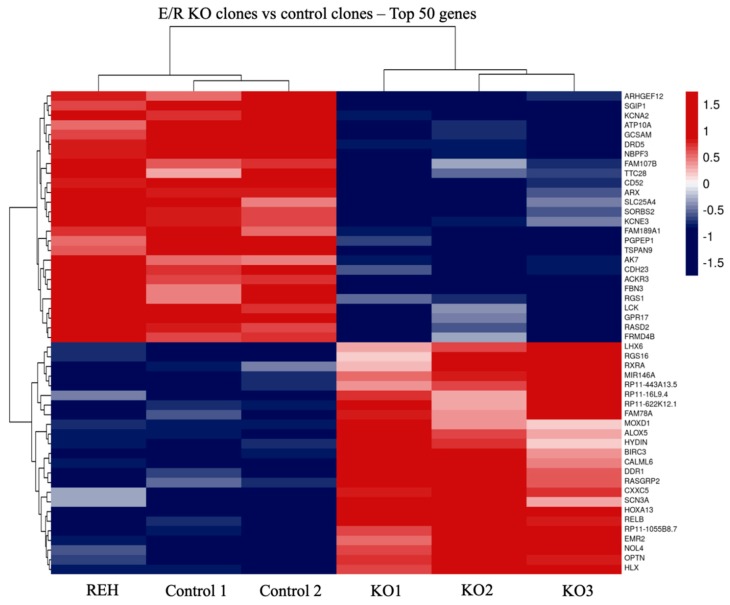
Transcriptomic analysis of E/R KO clones. Heat map of TOP50 differentially expressed genes in E/R Knock-out (KO) clones as compared with REH cells and control clones. Each row represents one differentially expressed gene; each column represents one clone. The dendrogram on the top reveals the sample clustering; the dendrogram on the left reveals the gene clustering.

The gene expression profile of E/R KO clones versus REH cells and controls clones, analysed by total RNA-sequencing, showed a total of 342 genes differentially expressed (q < 0.05), 182 upregulated and 160 downregulated (Table S3). The heatmap of the top50 of the most deregulated genes according to fold change (FC) values showed a distinct expression signature of E/R KO clones as compared with REH cells and control clones (Figure 2).

In order to elucidate the effect of *E*/*R* fusion gene abrogation on a functional level, the significantly deregulated genes were grouped into biological processes according to their function by enrichment analysis (Table S4 and Figure S3A). These biological processes were classified into 11 cluster representative: Germinal centre formation, regulation of response to external stimulus, positive regulation of multicellular organism process, negative regulation of apoptotic/necroptotic process, regulation of GTPase activity, I-kappaB kinase/NF-kappaB signalling, cellular calcium ion homeostasis, regulation of protein localization, regulation of cell adhesion, cytoskeleton organization and actin filament-based process (Figure S3B).

Some of these biological processes include genes whose deregulation has been described in ALL patients [21,22,23,24,25,26]. Among them we observed a downregulation of *CXCR7*, *LCK*, *PTPRG*, *VPS34*, *PTPRK, ARHGEF12*, *RGM* and *HAP1* genes and an upregulation of *RXRA*, *CXXC5*, *ARX*, *SORBS2*, *RGS16*, *TLR7*, *mir-146* and *TP63* genes. It is worth mentioning that *RGS16* and *PTPRK* genes are involved in PI3K/Akt/mTOR pathway, and *mir-146* and *TP63* are involved in the regulation of apoptosis. Both biological processes were proposed as key factors in maintenance of the oncogenicity of E/R-positive cells.

### 3.3. E/R Abrogation Reduces Proliferative Capacity and Resistance to Apoptosis In vitro

**Figure 3:**
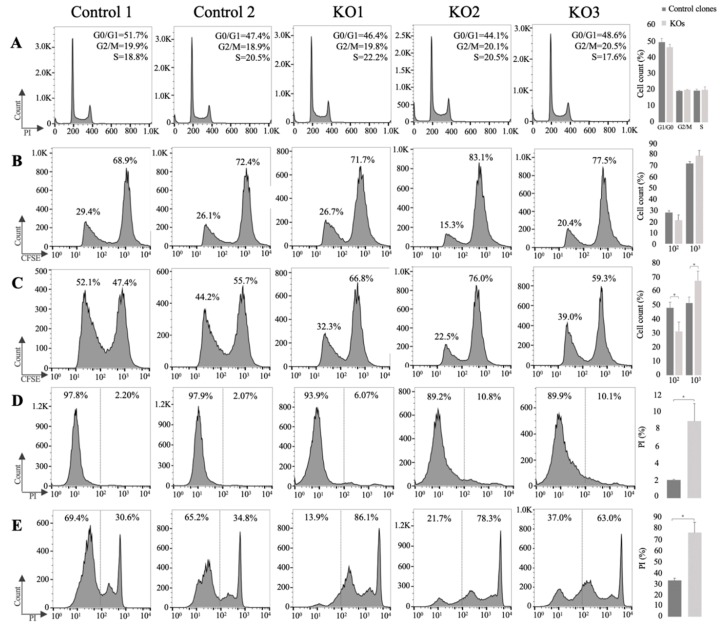
In vitro functional studies after E/R abrogation. (**A**) Cell cycle distribution of control clones and E/R KO cells at 48 h. (**B**) Carboxyfluorescein Succinimidyl Ester (CFSE) quantification by flow cytometry after 48 in culture. The peak on the right (10^3^) represents the percentage of cells that have not divided and the left peak (10^2^) represents the percentage of cells that have divided and therefore diluted their CFSE expression. (**C**) CFSE expression by flow cytometry of cells co-cultured with Mesenchymal Stromal Cells (MSC) cell line HS-5 at 48 h. (**D**) Apoptosis level quantification by Propidium Iodide (PI) expression. The figure shows the percentage of PI negative cells (left) and PI positive cells (right) at 48 h. (**E**) Apoptosis level quantification by PI expression after treatment with Vincristine (1 μM) at 48 h. On the right is represented the mean distribution of control clones (dark grey) and E/R KO clones (grey) of different experiments. All the experiments were carried out by triplicate. * *p* ≤ 0.05 (unpaired *t*-test).

To elucidate the biological effects of abrogation of E/R expression in the KO clones, functional studies were performed. MTT proliferation studies were performed at 24 h intervals up to 240 h. The results showed no proliferation differences between KO clones and REH cells or control clones (Figure S4). We simultaneously analysed the cell cycle distribution of the different cells by permeabilization followed by PI staining. No differences were observed between the different clones (Figure 3A). Moreover, no significant differences were observed through the expression of CFSE by flow cytometry (Figure 3B). By contrast, E/R KO clones showed a significantly lower proliferation rate than control clones when they were co-cultured with MSCs (HS-5) (*p* < 0.05) (Figure 3C).

**Figure 4:**
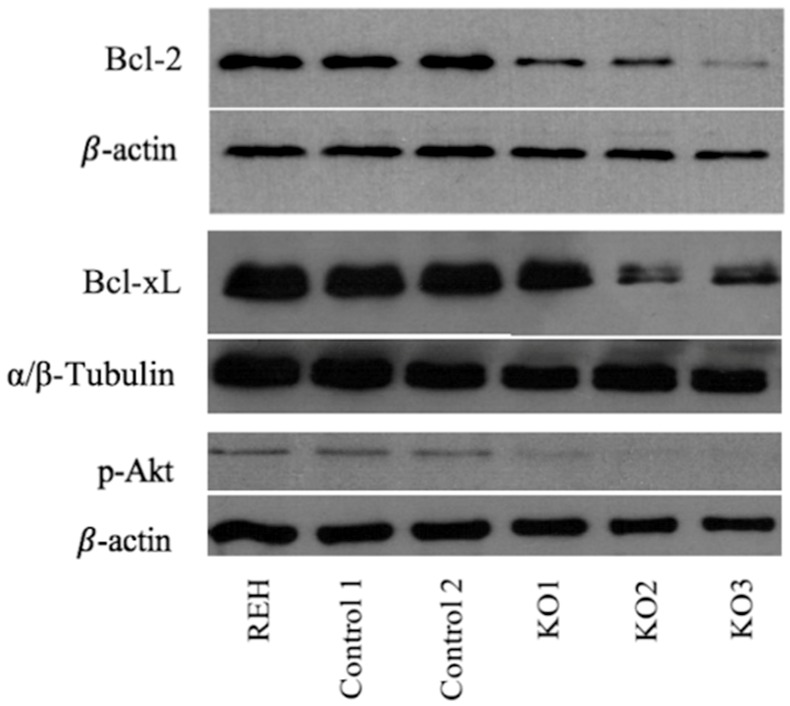
Western blot analysis of E/R targets expression. Lower phospho-Akt (60 kDa), BCL-XL (30 kDa) and BCL-2 (28 kDa) expression levels were observed in all E/R KO clones compared with parental cell line (REH) and control clones. This experiment had three replicates.

Deregulation of genes such as *miR-146a* or *TP63* observed by expression analysis suggested the alteration of cellular processes such as the regulation of apoptosis. Levels of anti-apoptotic factor such as *Bcl-2* or *Bcl-xL* gene have shown to play a key role in the survival of E/R-positive cells, protecting from programmed death [27,28]. To check these findings, Bcl-2 and Bcl-xL expression levels were measured through WB. Suppression of the fusion protein produced a decrease of 60% and 47% in the expression of Bcl-2 and Bcl-xL proteins respectively (*p* = 0.003; *p* = 0.043), thus reducing the resistance to apoptosis provided by the antiapoptotic factors of this family (Figure 4). In agreement with this observation, we detected an increase in the late apoptotic levels assessed by propidium iodide staining in E/R KO clones as compared with control clones (8.99 ± 2.08 vs. 2.135 ± 0.065) (*p* < 0.05) (Figure 3D). Treatment with Vincristine (1 μM) induced a greater late apoptotic rate in E/R KO clones as compared with control clones (75.8 ± 9.59 vs. 32.7 ± 2.1) (*p* < 0.05) (Figure 3E).

### 3.4. Abrogation of ETV6/RUNX1 Expression Enhances Sensitivity to the PI3K Inhibitor Copanlisib

Deregulation of *RGS16* or *PTPRK* genes also suggested the alteration of the PI3K/Akt/mTOR pathway. Several studies have already suggested that E/R may be key in the maintenance of the leukemic phenotype through the activation of different pathways, including the PI3K/Akt/mTOR pathway, resulting in proliferation and cell survival of leukemic cells. Akt phosphorylation levels measured through WB showed a reduction of 90% in Akt activity in the KO clones relative to REH cells and control clones (*p* = 0.003), suggesting the decrease in PI3K/Akt/mTOR activity as a result of the elimination of the expression of E/R (Figure 4).

A large proportion of relapsing E/R-positive patients become resistant to GCs such as Prednisolone, widely used in ALL treatment [8] and previous studies demonstrated that the use of PI3K inhibitors can sensitize E/R-positive cells to GCs [14].

**Figure 5:**
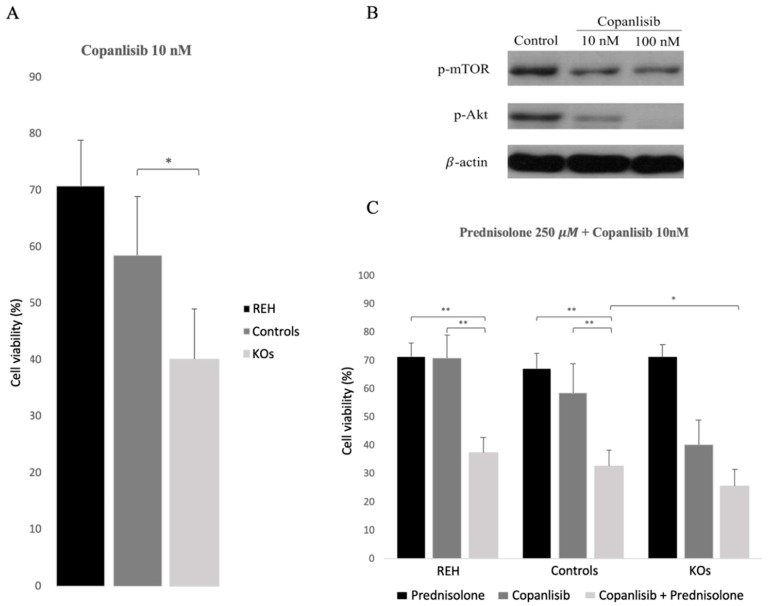
Cell viability and protein expression measured after Copanlisib/Prednisolone treatment. (**A**) Cell viability was measured by the 3-(4,5-Dimethyl-2-thiazolyl)-2,5-diphenyl-2H-tetrazolium bromide also named as Methylthiazolyldiphenyl-tetrazolium bromide (MTT) proliferation assay after treatment (192 h) with Copanlisib (10 nM). E/R KO clones (light grey square line) showed a higher sensitivity to Copanlisib than REH cells (black square line) and control clones (grey square line). This graph represents the average of three independent experiments and in turn the average of the 2 control clones and the 3 KO clones. (**B**) p-Akt (60 kDa) and p-mTOR (289 kDa) expression levels decreased after treatment with Copanlisib. (**C**) Prednisolone (black square line), Copanlisib (dark grey square line) and Copanlisib plus Prednisolone combination (grey square line) were tested in the different clones. The relative cell viability was calculated as the percentage of untreated cells. This experiment had three replicates. * *p* ≤ 0.05; ** *p* ≤ 0.005 (unpaired *t*-test).

After verifying a lower activation levels of PI3K/Akt/mTOR pathway with the elimination of E/R expression, we aimed to test if these cells responded in the same way to PI3K inhibitors. For that, we used Copanlisib, a PI3K inhibitor with inhibitory activity predominantly against the PI3K-alpha and PI3K-delta isoforms [29,30]. Treatment with Copanlisib (10 mM) resulted in higher decrease of viability in E/R KO clones compared with REH cells and controls clones (Figure 5A). To verify if Copanlisib was actually inhibiting the PI3K/Akt/mTOR pathway, we measured the phosphorylation levels of Akt and mTOR by WB, before and after treatment. We observed that the phosphorylation levels of both proteins decreased after treatment (Figure 5B).

On the other hand, treatment with Prednisolone (250 μM) was comparable to the effect of Copanlisib on E/R-positive cells. We did not observe a higher decrease of cell viability in E/R KO clones as compared with REH cells and control clones (Figure 5C). The combination of Copanlisib (10 nM) and Prednisolone (250 μM) showed a synergistic effect by decreasing the cell viability in REH and control clones expressing *E*/*R* fusion gene as compared with Prednisolone and Copanlisib alone. Furthermore, we also observed greater reduction of cell viability in E/R KO clones as compared with REH cells and control clones (Figure 5C).

### 3.5. E/R Repression Impairs the Tumourigenicity In vivo

**Figure 6:**
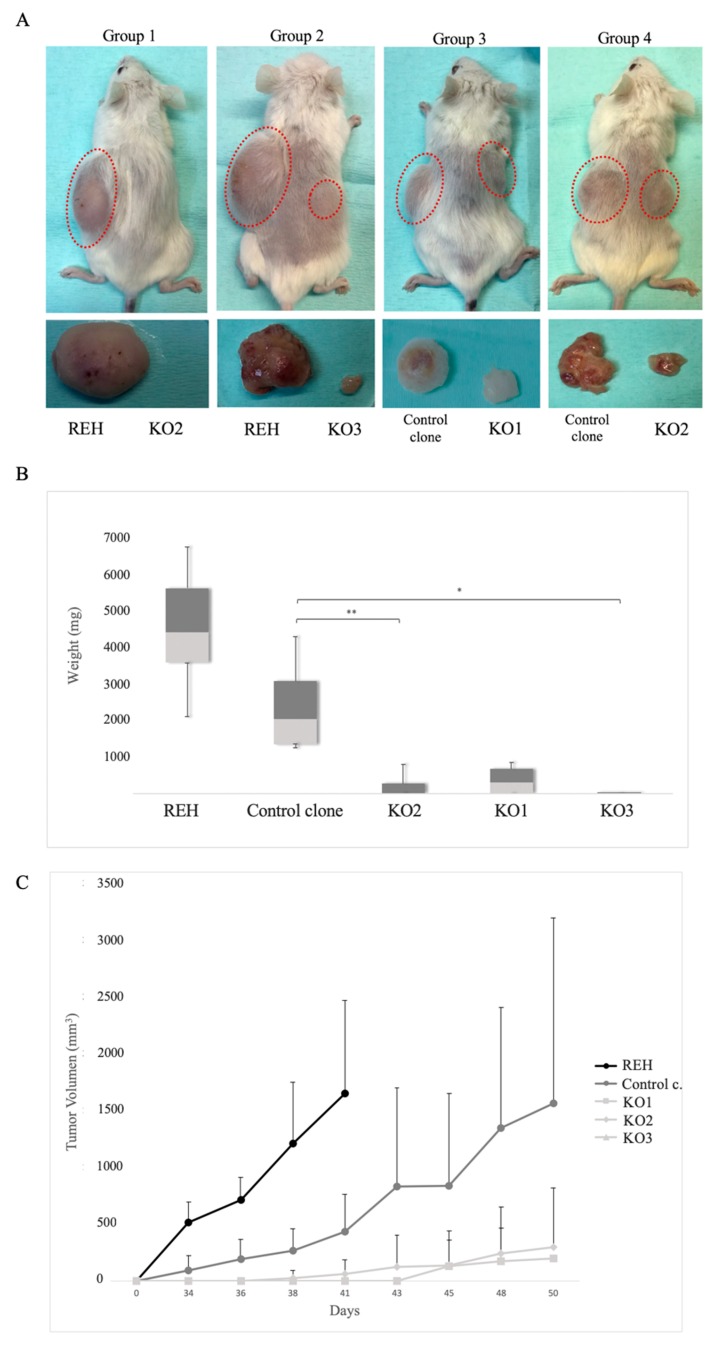
In vivo effects of CRISPR-mediated editing of the *E*/*R* oncogene. (**A**) External appearance of mice and developed tumours 48–62 days after subcutaneous cell injection. Tumours formed by KO clones (right flank) were smaller than those induced by REH cells or control clones (control c.) (left flank). Each group had four mice. (**B**) Evolution of tumour growth measured every 2–3 days until the moment in which mice were sacrificed. (**C**) Representation of the mean tumour size corresponding to each clone, independently of the group. * *p* ≤ 0.05; ** *p* ≤ 0.005 (unpaired *t*-test).

In order to determine the effects of E/R expression abrogation in vivo, 16 NSG mice were subcutaneously injected with REH cells or control clone (left flank) and KO clones (right flank). Only 6 mice injected with KO clones developed tumour growth on the right flank (6/16), whereas all those injected with REH or control clone developed a tumour (16/16). In the first group (REH cells vs. KO2 clone), none of flanks injected with KO2 developed tumour (mean mass: 0 mg ± 0 vs. 4872.5 mg ± 1323; *p* = 0.029). In the second group (REH cells vs. KO3 clone), only one of flanks injected with KO3 developed a tumour (1/4). This tumour was significantly smaller than those generated from REH cells (mean mass: 40 mg ± 69.3 vs. 4212.5 mg ± 1663.9; *p* = 0.029). In the third group (control clone vs. KO1), we observed tumour growth in 2/4 flanks of mice injected with KO1. These tumours were significantly smaller than those generated from control clone (mean mass: 483 mg ± 354.4 vs. 2470 mg ± 872.5 vs. *p* = 0.041). In the same way, 3/4 mice develop tumours from KO2 in the group 4 (control clone vs. KO2), but these tumours were significantly smaller than those generated from control clone (mean mass: 355 mg ± 293.6 vs. 2255 mg ± 1215.6; *p* = 0.029) ( Figure 6A and Figure S5). In general, subcutaneous tumours generated from E/R KO cells were significantly smaller than those produced by REH cells or control clone (mean mass 202 mg ± 298.9 vs. 4542.5 mg ± 1539, *p* < 0.001; 202 mg ± 298.9 vs. 2347.1 mg ± 1087.2, *p* ≤ 0.001) (Figure 6B).

In addition, significant differences were observed in the time of appearance of the tumours. Those generated through the KO clones appeared around day 42 (mean: 46.5 ± 7.8), unlike those generated by the REH cells or the control clone, which appeared around day 29 (mean: 29 ± 4.2; *p* = 0.001) and day 36 (mean: 36.6 ± 5.34; *p* = 0.03) respectively (Figure 6C).

Histopathological analysis of representative tumours from each group of mice revealed a proliferation of immature cells with a diffuse pattern (emerging from the dermis) infiltrating the muscle tissue and subcutaneous tissue (dermis and hypodermis) in tumours from REH cells and control clones. These tumours were larger than E/R KO tumours, showing some areas of apoptosis and necrosis. They were composed by round-shaped monoform cells with round nuclei and nucleoli. E/R KO tumours were smaller showing a nodular-diffuse pattern. These tumours were composed of monomorphic and more mature-appearing cells. These cells did not show nucleoli.

Tumours from REH and control clones revealed a higher number of mitotic figures as compared to tumours from KOs clones (52 vs. 20, *p* = 0.017//62 vs. 20; *p* = 0.006). Interestingly in KOs tumours, but not in REH and control clone tumours, we observed the “starry skyȍ (macrophages containing dead apoptotic tumour cells) (Figure S6). No other morphological changes between tumours were observed.

## 4. Discussion

In this study, we generated an E/R KO model in an ALL cell line carrying the t(12,21) in order to assess how the loss of fusion expression affects the tumour cells. Functional analysis by RNA-seq showed that the loss of fusion gene expression caused the deregulation of some genes involved in proliferation and apoptosis processes among others. In vitro, ALL cells showed a lower proliferation rate and a higher apoptosis sensitivity after *E*/*R* fusion gene abrogation. Furthermore, the loss of E/R expression was able to sensitize ALL cells to PI3K inhibitors. The xenograft model confirmed the results observed in vitro showing a lower oncogenicity of ALL cells after fusion gene suppression.

The recent evolution of genetic editing techniques with the CRISPR/Cas9 system has allowed, among others, the generation of KO gene models, helping us to better understanding the biology of diseases such as ALL [31]. We used CRISPR/Cas9 system to completely eliminate the expression of the E/R fusion protein.

The loss of fusion expression was checked, observing an absence of mRNA coding for E/R in the KO2 and KO3 clones and a leaky expression in KO1 clone. We hypothesize that this loss of mRNA was due to nonsense-mediated mRNA decay mechanism [32]. The design of the RT-qPCR oligonucleotides outside the editing region ruled out the expression of an aberrant form of the fusion protein between exon 5 of *ETV6* and exon 4 of *RUNX1*. Furthermore, the loss of E/R expression was observed by total RNA-seq in E/R KO clones. In this way we generated a powerful model on which to study the effect of the elimination of the fusion gene on leukemic cells. These cells maintain a series of secondary alterations that triggered the leukaemia, similar as occurs in patients.

Transcriptome analysis of different clones showed a huge number of genes significantly deregulated after *E*/*R* abrogation. Some of them have been previously described in E/R fusion protein silencing studies, such as *CXXC5*, *ARX* and *SORBS2,* which supports these results. Moreover, a high number of upregulated genes were observed after fusion protein abrogation which agrees with the repressive activity of the *E*/*R* fusion gene [33,34]. Some of these genes have been previously described to be directly regulated by *RUNX1* transcript factor [35]. The most of genes significantly deregulated are involved in different biological processes such as germinal centre formation, regulation of response to external stimulus, positive regulation of multicellular organism process, negative regulation of apoptotic/necroptotic process, regulation of GTPase activity, I-kappaB kinase/NF-kappaB signalling, cellular calcium ion homeostasis, regulation of protein localization, regulation of cell adhesion, cytoskeleton organization and actin filament-based process. These results are in agreement with previous studies, in which was demonstrated the implication of E/R in the cellular processes that may be maintaining the leukemic state of the tumour cells [14,28,33,36]. Furthermore, in our study we observed the downregulation of genes such as *VPS34*, *PTPKR*, *ARHGEF12*, *RGM* and *HAP1*. These findings are in agreement with previous studies by Ross E. and Yeoh E. in which they described the upregulation of these genes in E/R positive patients [25,26].

Within of the significantly deregulated genes we observed in our study the deregulation of genes involved in regulation of cell proliferation such as *RGS16* and *PTPRK* genes. *RGS16* who plays an antiproliferative role through inhibition of the PI3K/Akt/mTOR pathway [37,38,39] and inhibition of cell migration [40], was upregulated in E/R KO cells. Meanwhile *PTPRK* gene was downregulated after E/R fusion gene abrogation, in line with what was observed in E/R-ALL patients in which it was upregulated [25,26].

Very subtle changes were observed in cell proliferation and cell cycle distribution after *E*/*R* abrogation in vitro, in agreement with previous studies [12,14]. However, E/R KO clones showed a significantly lower proliferation rate when they were co-cultured with MSC. Mesenchymal cells have been shown to play a key role in the development and evolution of ALL [41,42] and MSCs induced greater cell adhesion, higher proliferation ratio and greater migration capacity to REH cells in a recent study [43]. Our data show that the *E*/*R* fusion gene therefore participates in the interaction of leukemic cells with the microenvironment and the loss of *E*/*R* fusion gene expression reverts the proliferative capacity that MSCs confer on leukemic cells.

The regulation of programmed death was another of the altered processes after the elimination of the fusion gene. In particular, an overexpression of the *mir-146* gene was observed. *miR-146* can regulate the expression of the apoptosis factor *STAT1*, and the anti-apoptosis factor *Bcl-xL*, thus promoting the apoptosis of ALL cells [44]. Furthermore, tumour protein P63 gene (*TP63* gene) was also upregulated in E/R KO clones as compared with control clones. This gene is involved in an antiapoptotic pathway that regulates the normal survival of B cells [45,46]. The decreased expression of antiapoptotic factors such as, Bcl-2 and Bcl-xL supported the observed results. E/R KO clones also showed a higher late apoptotic rate in vitro, demonstrating that the fusion gene regulates the expression of antiapoptotic factors that protect leukemic cells from apoptosis. Death levels were also higher in E/R KO clones after treatment with Vincristine.

Furthermore, we wanted to analyse whether the non-expression of E/R and consequently the loss of activation of the PI3K/Akt/mTOR pathway, was able to sensitize the cells to PI3K inhibitors. The use of PI3K inhibitors alone has shown to be an effective treatment in E/R-positive cells. In addition, the activity of these inhibitors in combination with Prednisolone, a GC widely used in the treatment of ALL, decreasing the resistance offered by E/R-positive cells to GCs. In our study, we observed that the use of Copanlisib, a PI3K inhibitor, achieved a significantly decrease of cell viability in E/R KO clones as compared with E/R-positive cells. We also observed that treatment with Copanlisib achieved the sensitization to Prednisolone in E/R-positive cells as recently described [14]. However, in our study, this sensitization was even higher in E/R KO cells. In this way, we can conclude that the fusion gene may be a good therapeutic target with which to improve the drug sensitivity of E/R-positive cells.

Finally, we wanted to check if E/R abrogation also decreased the tumour potential of cells in vivo. For that, a xenograft model was generated by injecting these cells into immunosuppressed mice, taking the injection of REH cells or a control clone on the opposite flank as control. Mice injected with KO clone cells did not generate tumours or generated smaller tumours than those generated by REH cells or control clone. The higher rate of mitotic activity in REH and control tumours observed through the histopathology analysis explains the greater growth of these tumours and reveals a greater tumoural capacity of these cells carrying *E*/*R* fusion gen in vivo.

Together these data show that by eliminating E/R expression, the cells lost tumourigenicity, decreasing its proliferative capacity, resistance to apoptosis and becoming more sensitive to PI3K inhibitors. Therefore, *E*/*R* fusion gene seems to play a key role in the maintenance of the leukemic phenotype, protecting cells from apoptosis and generating resistance to treatments. Furthermore, these data suggest that microenvironment confers a proliferative advantage to leukemic cells through E/R fusion gene. However, this study is based on a single E/R cell line, so it would be of great interest to reproduce these results in another cell line carrying the fusion gene to confirm these findings. In this way, although more studies are needed to elucidate the mechanism of action of the fusion gene and despite the good clinical course of these patients, this study suggests that the fusion gene could be a possible therapeutic target to design new drugs that prevent the expression of this protein, especially in those cases of relapse or lack of response to treatments.

## Supplementary Materials

The following are available online at https://www.mdpi.com/2073-4409/9/1/215/s1. Table S1: sgRNA designed against E/R fusion sequence, Table S2: Possible off-targets of the sgRNAs, Table S3: Genes significantly deregulated after E/R fusion gene abrogation, Table S4: Enrichment of differentially expressed genes after E/R abrogation, Figure S1: Edited-REH sequences, Figure S2: Edited sequences of single cell derived-cell line, Figure S3: GO Enrichment Analysis, Figure S4: MTT proliferation assay, Figure S5: Measure of tumour growth, Figure S6: Tumour growth and histopathological findings.
