# Supplementary material for: ETV6/RUNX1 Fusion Gene Abrogation Decreases the Oncogenicity of Tumour Cells in a Preclinical Model of Acute Lymphoblastic Leukaemia
Source: Cells. 2020 Jan 15;9(1):215. doi: 10.3390/cells9010215 (PMC7017301; doi:10.3390/cells9010215)
Supplement: Supplementary file 1 [file cells-09-00215-s001.pdf]

Supplementary information

ETV6/RUNX1 FUSION GENE ABROGATION DECREASE THE ONCOGENICITY OF TUMORS CELLS IN A PRECLINICAL MODEL OF ACUTE LYMPHOBLASTIC LEUKEMIA.

Adrián Montaño[1], Jose Luis Ordoñez[2], Verónica Alonso-Pérez[2], Jesús Hernández-Sánchez[1], Sandra Santos[1], Teresa González[3], Rocío Benito[1], Ignacio García-Tuñón*[2], Jesús María Hernández-Rivas[1,2,3,4]*

[1]IBSAL, IBMCC, Universidad de Salamanca-CSIC, Cancer Research Center; Salamanca; Spain.

[2]Unidad de Diagnóstico Molecular y Celular del Cáncer, Centro de Investigación del Cáncer-IBMCC (USAL-CSIC), Salamanca, Spain.

[3]Dept of Hematology, Hospital Universitario de Salamanca; Salamanca; Spain.

[4]Dept of Medicine, Universidad de Salamanca, Spain.

*These authors share senior authorship.

Corresponding author:

Jesús-María Hernández-Rivas

IBMCC, CIC Universidad de Salamanca-CSIC, Hospital Universitario de Salamanca

Paseo de San Vicente 58

37007 Salamanca

Spain

Phone: + 34 923291384 // Fax: +34 923294624

e-mail: jmhr@usal.es

Keywords

Acute lymphoblastic leukemia, ETV6/RUNX1, CRISPR/Cas9, Genome edition.

Supplementary tables and figures

Table S1. sgRNA designed against *E/R* fusion sequence. Two custom-designed single guide RNAs (sgRNAs) were designed to genetically inactivate the *E/R* oncogene. These specific sgRNAs direct Cas9 to the *E/R* fusion sequence. G1 sgRNA, directed at the end of exon 5 of *ETV6* and G2 directed to intronic region before the fusion point between *ETV6* and *RUNX1*.

	Forward	*Reverse*
G 1	caccgGCCTAATTGGGAATGGTGCG	aaacCGCACCATTCCCAATTAGGCc
G 2	caccgAAGAGCACGCCATGCCCATT	aaacAATGGGCATGGCGTGCTCTTc

Table S2. Possible off-targets of the sgRNAs. The possible off-targets of the sgRNAs used obtained from "Breaking Cas" website (http://bioinfogp.cnb.csic.es/tools/breakingcas/) were checked by PCR and sanger sequencing. Two pairs of oligonucleotides were designed for each off-target region.

Gen	*Score*	*Forward*	*Reverse*
PTPN21	0	AGTGTAATTTGGGAAACAGCCCT	TTGGGGCTTTTCCCACCTCAA
BCL9L	0.3	AGAGAATGGATCTGGGAGGGA	AGGCGAGGCAGTTGCAGTGTA
BEND4	0.1	GGCATCAGGTAGCCAACGTTC	CAGGATCAATGGATTTGTCACAG
VSIR	0	AAAGGGTCCAGAGAAGAGAGG	CTGGGGACGGAGCAAAACTTT

Table S3. Genes significantly deregulated after *E/R* fusion gene abrogation. List of 342 genes significantly deregulated after *E/R* fusion gene abrogation sorted according to the decreasing value of the FC. The TP50 of deregulated genes are shaded in grey. In the following columns from left to right, the mean of normalized counts of all samples (baseMean); log2-Fold Change (log2FC); log2FC unshrunk, P-value and adjusted P value.

Gene	baseMean	log2FC	log2FCunshrunk	Pvalue	Padj
DRD5	154.4	-2.34959	-3.92883	0	0
ATP10A	231.1	-2.07428	-3.35631	0	0
SGIP1	70.3	-2.0645	-3.86631	0	0
BIRC3	80.1	1.98755	4.09318	0	0
ACKR3	66.8	-1.98193	-3.84476	0	0
EMR2	80.4	1.97712	3.98976	0	0
FAM189A1	55.5	-1.80486	-4.18028	0	1.97E-13
MIR146A	278.6	1.77216	2.54174	0	1E-15
OPTN	315.4	1.7413	2.24832	0	0
LHX6	127.2	1.72519	3.8055	4E-15	3.17E-12

NBPF3	35.4	-1.67532	-4.9272	1.6E-14	1.23E-11
GCSAM	1072	-1.67221	-1.90226	0	0
PGPEP1	94.7	-1.66199	-3.62221	3.7E-14	2.72E-11
KCNA2	143.1	-1.65663	-2.17481	0	1E-15
RP11-622K12.1	367.2	1.59169	2.28594	2E-15	1.94E-12
RASD2	209.2	-1.47352	-2.0491	6.1E-14	4.04E-11
CDH23	265.2	-1.44672	-1.96584	6E-14	4.04E-11
ALOX5	89.6	1.37691	2.54469	2.65E-10	1.38E-07
SORBS2	882	-1.364	-1.63045	0	4.4E-14
HYDIN	83.6	1.36022	3.17093	5.3E-10	2.67E-07
GPR17	119.4	-1.33855	-2.68649	1.05E-09	4.79E-07
MOXD1	162.7	1.33314	3.05082	1.17E-09	5.15E-07
ARX	64.4	-1.30499	-2.44801	2.28E-09	9.76E-07
KCNE3	249.5	-1.30368	-1.95828	2.64E-10	1.38E-07
HOXA13	234.3	1.29849	1.52883	0	1.13E-13
LCK	214.4	-1.29552	-2.04607	7.9E-10	3.84E-07
RELB	271.2	1.28523	1.51994	0	4.11E-13
DDR1	207.4	1.23577	1.58447	9.25E-12	5.62E-09
SCN3A	361.8	1.23448	1.91793	3.86E-09	1.52E-06
FRMD4B	807.8	-1.22731	-1.62866	8.07E-11	4.52E-08
LIMS2	495.1	-1.20713	-2.10548	2.5E-08	7.02E-06
RGS1	221.1	-1.19302	-1.85261	1.24E-08	4.03E-06
CALML6	75.1	1.19083	1.84701	1.21E-08	4E-06
FZD3	83	1.179	2.86812	6.42E-08	1.49E-05
ARHGAP42P2	187.8	-1.17718	-2.04583	5.34E-08	1.25E-05
RP11-251M1.1	558.7	1.1701	1.89706	3.88E-08	9.91E-06
RXRA	695.7	1.16993	1.68558	6.77E-09	2.47E-06
RGS16	431.5	1.16993	4.27179	1.85E-08	5.52E-06
HOTTIP	40.8	1.16894	2.4038	1.01E-07	2.26E-05
FBN3	196.1	-1.16298	-1.45832	3.86E-11	2.25E-08
RP11-16L9.4	439.2	1.15288	1.66285	1.14E-08	3.86E-06
HLX	101.3	1.14724	1.59611	5.25E-09	1.97E-06
TP63	318.4	1.1262	1.88183	1.55E-07	3.14E-05
TLR7	61.4	1.12406	2.8608	2.25E-07	4.37E-05
PTPRG-AS1	73.4	-1.11862	-3.58436	1.17E-07	2.44E-05
FAM78A	548.8	1.11483	1.43424	9.77E-10	4.59E-07
BCAS1	210.8	-1.11072	-1.8555	2.28E-07	4.38E-05
PLS1	62.8	1.10389	2.20117	4.87E-07	8.16E-05
AQP5	229.5	1.09449	1.83256	3.47E-07	6.1E-05
PDE10A	95.9	-1.08807	-2.11786	6.94E-07	0.000109
CSF1	29.6	1.07667	3.30357	4.18E-07	7.16E-05
TTC28	422.8	-1.06872	-1.42756	1.9E-08	5.53E-06
EPHB6	332.1	1.05097	1.40655	3.42E-08	8.9E-06
GIMAP6	104.7	-1.04917	-2.08309	1.74E-06	0.000237
CNR2	151.2	-1.04836	-1.46725	1.15E-07	2.43E-05
SGK1	203.2	1.04539	1.4315	7.51E-08	1.71E-05

TRPM2	307.5	1.04389	1.56381	4.22E-07	7.16E-05
SLC25A4	375.9	-1.04233	-1.35587	1.71E-08	5.4E-06
SERPINB1	449.6	-1.03395	-1.48499	2.89E-07	5.4E-05
ADCY7	789.1	1.02767	1.67585	1.47E-06	0.000206
EPN2	211.7	-1.02564	-2.01505	2.94E-06	0.000382
NPL	61.1	1.01372	1.61799	1.64E-06	0.000226
RP11-1055B8.4	129.1	1.00869	1.41433	3.47E-07	6.1E-05
CD52	5748.7	-1.00638	-1.07155	0	0
C10orf10	193.8	-1.00598	-1.707	3.06E-06	0.000394
MYO7B	130	-1.00505	-2.57867	3.37E-06	0.000419
N4BP3	1631.4	1.0044	1.34839	1.49E-07	3.05E-05
TRNP1	843.7	-1.00257	-1.36303	2.17E-07	4.27E-05
RP11-443A13.5	571.4	1.00152	1.21962	2.54E-09	1.06E-06
LRP4	64.7	0.99177	2.46045	4.97E-06	0.000603
TSPAN9	526.5	-0.99105	-1.11836	1.14E-12	7.23E-10
TMEM64	260.3	0.98891	1.45127	1.27E-06	0.000182
TCF7	151	-0.98381	-1.32807	2.96E-07	5.46E-05
MMP14	192.5	0.98032	1.80139	7.14E-06	0.000846
FAM107B	10387.3	-0.97505	-1.19805	1.14E-08	3.86E-06
PLEK	1078.2	0.96863	1.28742	3.1E-07	5.65E-05
NOL4	214.5	0.96397	1.17625	1.07E-08	3.8E-06
CAV1	297	-0.95749	-1.27142	4.08E-07	7.08E-05
AGR2	38.8	0.95646	5.4769	9.71E-07	0.000149
RGMA	1017.8	-0.95634	-1.18978	4.04E-08	1.02E-05
RASSF4	289	-0.95437	-1.75826	1.25E-05	0.001381
LCN6	249.8	0.95321	1.81714	1.36E-05	0.001474
RP11-1055B8.7	6983	0.94416	1.00376	0	0
PTMS	72.4	0.94242	1.9579	1.77E-05	0.001864
CYTH4	524.2	0.9396	1.24137	5.87E-07	9.41E-05
KLHDC7B	22.2	0.93929	2.83172	9.63E-06	0.001105
HAP1	3579.1	-0.936	-1.13741	2.31E-08	6.59E-06
RHOH	1146.9	-0.9267	-1.23334	1.02E-06	0.000153
IRF8	803.1	-0.92338	-1.25995	1.95E-06	0.000263
LARGE	415.8	-0.91806	-1.3959	1.02E-05	0.001156
LAMB4	79.9	-0.9156	-1.67425	2.72E-05	0.002662
AK7	1333.1	-0.91269	-1.0905	1.8E-08	5.52E-06
TBXAS1	369.3	0.9111	1.09726	3.12E-08	8.26E-06
PLEKHB1	67.2	0.90934	1.81052	3.42E-05	0.00322
SPTBN4	89.6	-0.90781	-1.24563	3.11E-06	0.000397
CXXC5	1769.6	0.90625	1.05615	3.28E-09	1.33E-06
GUCY1A3	222.9	-0.89857	-1.50261	2.9E-05	0.00282
GPR142	27.8	-0.89415	-2.85582	1.96E-05	0.001998
ZNF264	134.7	0.89389	1.26566	7.45E-06	0.000869
SNTA1	325.1	-0.88594	-1.09918	3.25E-07	5.86E-05
SV2A	972.4	0.88563	1.08285	1.7E-07	3.39E-05
ZNF469	158.6	0.88264	1.58403	5.07E-05	0.004447

PIGM	310.8	0.88148	1.10151	4.97E-07	8.23E-05
AC090044.2	95.8	0.87855	1.7981	6.3E-05	0.005364
SEMA7A	122.3	0.87808	1.47324	4.44E-05	0.004041
NTN3	154.2	0.87613	1.09977	6.78E-07	0.000107
LINC01013	150	-0.87602	-1.36521	3.06E-05	0.002955
CROCC	298.9	0.87521	1.03825	4.13E-08	1.02E-05
MMP15	449.3	-0.8698	-1.13171	2.58E-06	0.000344
RP11-1055B8.6	283	0.86919	1.226	1.27E-05	0.001392
EMP2	378.1	0.86759	1.09257	9.99E-07	0.000152
RP11-431N15.2	20.1	0.86663	5.26703	6.26E-06	0.000747
ZNF528	133.8	0.8636	1.48223	6.46E-05	0.005392
FCGR2A	66.4	0.86013	1.72152	8.92E-05	0.006923
NEIL1	67.7	0.85161	1.49228	8.63E-05	0.006797
SIT1	183.8	0.85063	1.50487	9.1E-05	0.006981
NT5E	323.3	-0.85004	-1.0867	2.66E-06	0.000352
CECR2	268.9	0.84865	1.17241	1.5E-05	0.001603
SERPINB6	560.5	-0.84378	-1.0843	3.67E-06	0.000453
SIDT1	263.9	-0.84107	-1.39962	8.96E-05	0.006923
AFF2	979.3	0.83909	1.10828	8.18E-06	0.000947
TEAD3	73	0.83854	1.4096	9.67E-05	0.007272
CD302	207.1	0.83694	1.39459	9.73E-05	0.007272
C1QTNF4	454.5	-0.83462	-1.02567	1.03E-06	0.000153
PTPN7	496.1	-0.83337	-1.42407	0.000115	0.008254
LBH	1252.6	-0.8326	-1.02589	1.22E-06	0.000178
RIC3	24	0.83244	4.0662	1.94E-05	0.001998
SEMA4A	185.8	0.83085	1.17682	3.18E-05	0.003026
NRBP2	246.9	0.82947	1.18658	3.71E-05	0.00344
TSKU	93.4	0.82585	1.35517	0.000112	0.008122
CD40	168.6	0.82537	1.01695	1.47E-06	0.000206
DBNDD1	403.2	-0.82471	-1.16418	3.51E-05	0.00328
KCNJ4	36.7	-0.82267	-1.86593	0.000169	0.011166
SORBS1	27.8	0.81941	2.04647	0.000157	0.010539
IL4I1	31.7	0.81657	2.62006	8.98E-05	0.006923
RP11-469H8.6	67.9	0.81609	2.12138	0.000151	0.010331
GNG11	1694.7	-0.81545	-1.06571	1.16E-05	0.001309
CAP2	128.4	-0.81443	-1.23333	8.73E-05	0.006837
PLEC	410.6	0.80672	1.04502	1.17E-05	0.001309
PDZD7	100.6	0.80051	1.19706	0.000104	0.007603
TMEM52	235.2	0.79981	1.61531	0.00027	0.016797
AC090044.1	372.3	0.79949	1.33198	0.000198	0.01259
ATP13A2	773.7	0.79809	0.98318	3.28E-06	0.000412
NPY	354.1	0.79767	1.1838	0.000104	0.007611
SLC51A	923.8	-0.79604	-1.11872	6.19E-05	0.005302
PSD2	32.5	0.78983	1.91702	0.000279	0.017214
CAST	535.9	0.78854	1.09765	6.42E-05	0.005392
PRR5L	357.9	-0.785	-0.9029	1.1E-07	2.39E-05

ARHGEF12	926.6	-0.78172	-0.87228	5.27E-09	1.97E-06
C20orf197	21.9	0.78168	2.91678	0.000115	0.008254
ZNF704	1650.9	-0.78124	-0.89744	1.15E-07	2.43E-05
DAB2IP	529.9	0.77986	0.99995	1.79E-05	0.001872
TMEM173	173.5	0.77921	1.1944	0.00019	0.012234
IGHD	430.3	0.77753	1.17758	0.000182	0.011865
ACTN1	1971.1	0.7764	0.88027	4.2E-08	1.02E-05
RP3-455J7.4	112.6	0.7684	1.51577	0.000463	0.025573
TMEM229B	179.1	0.76807	1.38713	0.000428	0.024342
NAT8L	59	0.7666	1.47598	0.000471	0.02581
RBMS2	105.9	0.76601	1.02433	5.69E-05	0.004937
SPNS2	96.1	-0.76441	-1.13302	0.000197	0.01259
FAM65B	1931.9	-0.76332	-1.03087	7.24E-05	0.005959
KCTD12	44.2	0.7633	1.79551	0.00046	0.025497
LOXHD1	82	-0.76287	-2.05591	0.000363	0.021387
ARL4C	117.7	-0.7613	-1.60277	0.000521	0.027621
PDLIM1	3779.7	-0.75931	-1.07166	0.00014	0.009802
ZNF169	102.5	0.75727	1.06339	0.000135	0.009626
TPM2	503.6	0.75349	0.95242	2.39E-05	0.002421
PFN2	420.2	0.75328	1.19692	0.000376	0.022024
WFS1	101.5	0.75075	1.18653	0.000381	0.022203
LCN10	42.8	0.74986	1.967	0.000486	0.026329
LILRB4	30.2	-0.74518	-2.02167	0.000494	0.02656
NCALD	46.4	-0.74335	-1.48403	0.000709	0.034244
ID2	152.8	0.74327	1.53331	0.00071	0.034244
CAMK4	217.7	0.74297	0.96783	6.03E-05	0.0052
PDGFA	327.7	0.74222	1.18772	0.000473	0.025829
TEX14	53.7	0.74155	2.36737	0.000355	0.02103
SPRY1	230.1	-0.73794	-0.92144	2.53E-05	0.002512
STIM1	473.7	-0.73641	-0.97332	9.19E-05	0.00701
SPARC	101.2	0.7362	2.12186	0.000497	0.026604
CACNA2D1	75.1	0.73588	1.23955	0.000631	0.031553
ITGB2	713.9	0.73385	0.972	0.000101	0.007414
CTD-3018O17.3	68.5	0.73343	2.08948	0.000537	0.028172
ANO1	25.8	-0.73342	-1.94635	0.000632	0.031553
C20orf194	216.2	0.73325	0.93779	5.16E-05	0.004504
MS4A4A	134.3	0.73223	0.95342	7.59E-05	0.006175
GARNL3	45.5	0.73071	1.25472	0.000723	0.034641
CARD9	84.4	0.72843	1.27716	0.000792	0.037227
LINC00114	50.9	0.72708	1.56987	0.000912	0.041384
GALNT10	153.9	0.72678	1.17004	0.000634	0.031553
C1orf222	60.1	0.7267	3.38097	0.00016	0.010692
RASGRP2	5671	0.72471	0.80094	1.86E-08	5.52E-06
RP11-830F9.7	568.5	0.72421	0.97573	0.000161	0.0107
MAP3K15	84	0.7234	2.14555	0.000581	0.030165
TACC2	14.2	-0.7217	-2.80884	0.000312	0.018838

CORO2A	263.9	-0.72125	-1.07485	0.000465	0.025585
TNFAIP8	784.8	-0.72046	-0.80155	5.25E-08	1.25E-05
PKIG	180.6	0.7152	1.42286	0.001122	0.048384
TLR3	112	0.71488	1.14196	0.000752	0.035921
LMNA	28	0.71359	1.78526	0.000974	0.043658
TMPRSS15	176.6	-0.71246	-1.40711	0.001171	0.049885
FAM132B	448.9	-0.71207	-0.93437	0.000138	0.009751
LACC1	65.3	0.71038	1.2899	0.00113	0.048557
BRINP2	106.2	-0.70892	-1.81139	0.000999	0.044262
HNRNPA1P37	148.6	-0.70868	-1.18719	0.000988	0.04402
FAIM3	190.7	-0.70773	-1.18642	0.001006	0.044414
ZNF641	98.1	0.70696	1.05068	0.000587	0.030165
TTC9	223.9	-0.7064	-1.07029	0.000674	0.032979
ABLIM1	225.1	-0.70592	-1.07602	0.000704	0.034244
C10orf128	142.5	0.70535	1.8123	0.00105	0.045788
BTN2A3P	99.4	0.69936	1.01969	0.000588	0.030165
PTPN3	66.8	-0.69834	-1.16774	0.001161	0.049594
SERINC5	435.8	-0.69699	-0.97851	0.000449	0.02528
SLC38A1	6284.3	-0.69645	-0.87412	8.05E-05	0.006483
DAPK2	666.3	-0.69437	-0.83711	2.59E-05	0.002554
GIMAP8	19.7	-0.69337	-2.63213	0.000536	0.028172
BMP2K	1727.1	-0.69233	-0.75947	2.78E-08	7.64E-06
HIP1R	570	-0.6917	-0.84738	4.71E-05	0.004258
HTRA3	17.2	0.6915	2.51822	0.000634	0.031553
DSTYK	220	0.6907	0.87977	0.000126	0.009033
BCL11B	316.1	0.68927	0.99353	0.000652	0.03221
PLXNC1	284.8	0.68588	0.78584	2.71E-06	0.000356
ORAI2	1167.4	-0.68344	-0.88109	0.000189	0.012234
XBP1	5123.5	-0.68319	-0.93619	0.000456	0.025497
CDH24	1294	0.68212	0.84292	7.64E-05	0.006185
CD72	1662	0.68122	0.88181	0.000213	0.013462
PCDHGC4	364.2	-0.68111	-0.82387	4.12E-05	0.0038
GREB1	1076	-0.68088	-0.95199	0.000586	0.030165
KCNQ1OT1	78.4	0.67777	1.03449	0.001142	0.048945
MBOAT1	104.5	0.67352	1.00355	0.001076	0.046676
ZFHX3	434.9	0.67246	0.91572	0.00052	0.027621
NEAT1	2886.8	0.67221	0.95417	0.000787	0.037215
PCCA	700.6	-0.67141	-0.84344	0.000147	0.010087
LRRC4	495.5	-0.6704	-0.79996	3.38E-05	0.003202
BCL6	2112.8	-0.66649	-0.72574	2.96E-08	7.98E-06
PTPRK	5372.9	-0.66494	-0.75968	4.57E-06	0.000559
ALDH3A2	645	-0.66287	-0.85065	0.000271	0.016797
METRNL	199.1	-0.6628	-0.81304	9.94E-05	0.007392
RP11-715J22.6	155.9	0.66094	0.85659	0.000331	0.019844
FMO4	18.3	0.65854	2.85429	0.000706	0.034244
MFI2	975.1	0.65699	0.78398	4.86E-05	0.004367

CBR3-AS1	468.6	-0.65573	-0.7299	7.64E-07	0.000118
GALNT12	180.5	0.65362	0.79167	8.6E-05	0.006797
MECOM	542.8	-0.64174	-0.83026	0.000482	0.02623
GIPC1	309.8	0.6412	0.80317	0.000271	0.016797
SIKE1	1347.5	-0.63951	-0.85494	0.00079	0.037227
TAGLN3	209.4	-0.63932	-0.79078	0.000213	0.013462
AC002454.1	1316.6	-0.63931	-0.76796	9.63E-05	0.007272
AATK	11.4	-0.6371	-3.43338	0.000604	0.03067
ZC3H12A	275.7	0.63261	0.82339	0.000632	0.031553
MYOM2	241.4	0.63191	0.82335	0.000651	0.03221
LAX1	250.8	-0.61615	-0.79876	0.000827	0.038338
RMND5B	689.1	0.61304	0.75966	0.000402	0.023211
GPSM1	5511.7	0.60465	0.71777	0.000157	0.010539
SSH1	1022.2	-0.60434	-0.66309	1.27E-06	0.000182
DPEP1	659.7	0.59957	3.82265	0.000718	0.034544
PECR	528.8	-0.5978	-0.66384	5.37E-06	0.000646
AVEN	268.8	-0.59717	-0.75211	0.000767	0.036418
LIG4	1424.3	-0.59515	-0.76201	0.001043	0.045721
JAM2	167.8	0.59361	0.74641	0.000798	0.037408
C12orf23	3429.3	-0.59254	-0.63665	1.07E-07	2.36E-05
HLA-F	1579	0.59166	0.72213	0.00046	0.025497
TMEM169	243.2	-0.58601	-0.73804	0.00096	0.043177
PPM1L	569	0.58504	0.69388	0.000247	0.015533
GRAMD1B	666.5	-0.58441	-0.72068	0.000674	0.032979
TCL1A	14125.8	-0.58379	-0.7102	0.000508	0.027111
LTBP4	1986	-0.58366	-0.70366	0.000408	0.023403
GABRA3	600.3	0.58365	0.73228	0.000946	0.042792
CLSTN3	436.5	0.58028	0.65011	1.96E-05	0.001998
OPN3	765.2	-0.57808	-0.64306	1.25E-05	0.001381
INO80C	371.9	0.57505	0.70177	0.000659	0.03245
AJUBA	747	0.57157	0.65377	8.59E-05	0.006797
LRRC8C	2327.4	-0.57157	-0.70511	0.000891	0.040809
PIK3C3	6227	-0.56954	-0.63715	2.53E-05	0.002512
TSPAN7	257.4	0.56592	0.65737	0.000194	0.012467
ANXA2	1995	0.56427	0.62478	1.39E-05	0.001501
IL1RAP	795.7	-0.56395	-0.66952	0.000424	0.024203
NFKB2	609.9	0.56343	0.67862	0.000628	0.031553
BLVRB	781.5	-0.56098	-0.62694	3.12E-05	0.002991
IER2	3387.6	0.56098	0.64561	0.000153	0.010429
DENND1B	599.4	-0.55701	-0.67477	0.000829	0.038338
MAP4K5	1334.5	0.55277	0.59071	2.82E-07	5.33E-05
BCL3	644.3	0.55203	0.60895	1.56E-05	0.001661
LDB2	465.5	-0.5518	-0.6714	0.001019	0.044866
SMC6	3061.7	-0.54865	-0.62885	0.00018	0.01179
ZNF253	1646.3	-0.54837	-0.64474	0.00046	0.025497
NRN1	2783.7	-0.54527	-0.6447	0.000585	0.030165

SLC39A8	923.8	-0.54477	-0.65199	0.000815	0.037948
CIC	1809	0.54446	0.60198	2.46E-05	0.002474
SYNE3	1544.9	-0.54423	-0.60647	4.41E-05	0.004041
EVPL	980.6	-0.54063	-0.61483	0.000157	0.010539
METTL17	1465.2	-0.53989	-0.6443	0.000846	0.039027
PARVG	875.4	0.53972	0.62981	0.000445	0.025159
KLHL6	2008.1	-0.53792	-0.59875	5.01E-05	0.004447
ZNF772	554.5	0.53727	0.60117	7.17E-05	0.005934
KRT9	18.6	0.53498	6.65035	0.000955	0.043075
SETD5-AS1	404.9	0.53251	0.63055	0.000814	0.037948
IRS2	1062.6	-0.52974	-0.61918	0.000598	0.030595
TNFAIP3	10109.7	0.52792	0.56264	5.83E-07	9.41E-05
ASAP2	1227.8	-0.52222	-0.60451	0.000523	0.027621
HCP5	3037.7	0.52175	0.56108	3.25E-06	0.000412
EEF2K	859.8	0.52046	0.58695	0.000184	0.011958
PRKD2	2447.4	0.51983	0.59259	0.000309	0.018771
CTD-2368P22.1	306	0.51516	0.60814	0.001108	0.047917
ZNF70	727.1	0.50734	0.57554	0.000349	0.020819
RLTPR	4653.6	-0.5058	-0.53588	5.61E-07	9.19E-05
MYO1G	1804.3	-0.50459	-0.58325	0.000767	0.036418
TBC1D2B	1080.7	-0.50443	-0.5858	0.000894	0.040817
CORO1C	2814.4	-0.50423	-0.54246	7.24E-06	0.000851
ABHD4	962.1	0.50249	0.58571	0.001045	0.045721
TUBA1A	6080.4	-0.49898	-0.54946	8.35E-05	0.006682
XYLT1	2173.2	0.49304	0.55528	0.000372	0.021838
CAMTA2	666.3	0.488	0.54536	0.000291	0.017889
HERC3	923.4	-0.48726	-0.53755	0.000139	0.009751
PPP1R13B	1190.8	-0.48723	-0.53054	5.06E-05	0.004447
DARS	6357.7	-0.48207	-0.50954	1.17E-06	0.000172
HMGN5	399.5	0.48135	0.55117	0.000978	0.043733
RNPC3	603.8	0.47969	0.54177	0.000603	0.03067
NREP	12968.7	-0.47495	-0.53106	0.000433	0.024565
AHDC1	2906.2	0.47326	0.53736	0.000883	0.040572
STK24	4714.6	-0.47268	-0.4991	1.53E-06	0.000213
GNG2	3002.9	-0.46988	-0.5218	0.00035	0.020819
ACSS1	2289.9	-0.46887	-0.52175	0.000403	0.023211
LAT2	9833.8	0.46614	0.50333	4.94E-05	0.004418
MXRA7	1139.6	-0.45464	-0.49246	0.000101	0.007414
ADD3	2155.4	-0.45395	-0.48413	1.9E-05	0.00198
PRPSAP2	1515.8	-0.45205	-0.49128	0.000146	0.010068
KAZALD1	816.8	0.44361	0.48156	0.000177	0.011647
KIAA0930	1631	-0.442	-0.47432	6.48E-05	0.005392
CCDC117	2855	-0.44158	-0.47766	0.000141	0.009858
ZNF302	1339.8	0.43871	0.47895	0.000316	0.019048
MAPK13	1114.4	-0.43871	-0.48379	0.000588	0.030165
HLA-A	14689.3	0.42258	0.45102	7.51E-05	0.006147

ZDHHC3	1299.3	-0.41703	-0.45071	0.000304	0.018507
H2AFY	16915.2	-0.41676	-0.45876	0.000997	0.044262
PCBP1-AS1	1351.6	0.41247	0.44775	0.000489	0.02639
SSBP2	6589.7	-0.41056	-0.4359	6.44E-05	0.005392
SMARCA2	4420.1	0.40579	0.43315	0.000145	0.010068
BZRAP1	6827.9	0.38932	0.41596	0.000293	0.017946
ETS2	7236.6	0.38757	0.40978	9.69E-05	0.007272
IDH2	9359.9	-0.37641	-0.40659	0.001075	0.046676
UNG	4993.8	-0.32718	-0.3456	0.000909	0.041384
STK38	5328.8	-0.32423	-0.33965	0.000391	0.022703

Table S4. Enrichment of differentially expressed genes after *E/R* abrogation. The first column shows the altered biological processes, following by expected value, fold enrichment, raw P value and the False Discovery Rate (FDR).

GO biological process complete	Expected	Fold Enrichment	raw P value	FDR
T cell receptor V(D)J recombination	.08	39.62	1.74E-04	3.58E-02
Germinal center formation	.12	33.01	2.19E-05	1.05E-02
Negative regulation of necroptotic process	.18	22.01	7.68E-05	2.21E-02
I-kappaB kinase/NF-kappaB signaling	1.05	6.70	1.40E-04	3.20E-02
Regulation of interleukin-6 production	2.01	4.47	2.88E-04	4.75E-02
Cellular calcium ion homeostasis	6.47	3.09	1.43E-05	7.53E-03
Regulation of inflammatory response	4.98	3.01	2.23E-04	4.05E-02
Regulation of lymphocyte activation	7.72	2.72	5.35E-05	1.69E-02
Regulation of GTPase activity	7.21	2.64	1.78E-04	3.56E-02
Regulation of cell migration	12.53	2.55	2.06E-06	2.50E-03
Actin filament-based process	8.42	2.49	1.71E-04	3.60E-02
Regulation of cellular protein localization	8.04	2.49	2.52E-04	4.37E-02
Neuron projection development	9.98	2.40	1.30E-04	3.12E-02
Cell morphogenesis	10.56	2.37	1.07E-04	2.65E-02
Positive regulation of immune response	12.77	2.19	1.46E-04	3.30E-02
Negative regulation of apoptotic process	13.48	2.15	1.39E-04	3.24E-02
Positive regulation of developmental process	20.46	2.15	2.91E-06	2.87E-03
Positive regulation of multicellular organismal process	26.05	2.07	5.37E-07	1.70E-03
Cytoskeleton organization	16.12	2.05	1.54E-04	3.35E-02
Negative regulation of response to stimulus	24.14	2.03	3.45E-06	3.21E-03
Regulation of anatomical structure morphogenesis	16.05	1.99	2.70E-04	4.54E-02
Positive regulation of molecular function	26.79	1.75	2.29E-04	4.11E-02
Regulation of signal transduction	49.65	1.51	2.44E-04	4.29E-02
Positive regulation of cellular process	80.91	1.37	2.11E-04	3.88E-02
Unclassified	48.68	.49	5.25E-05	1.77E-02

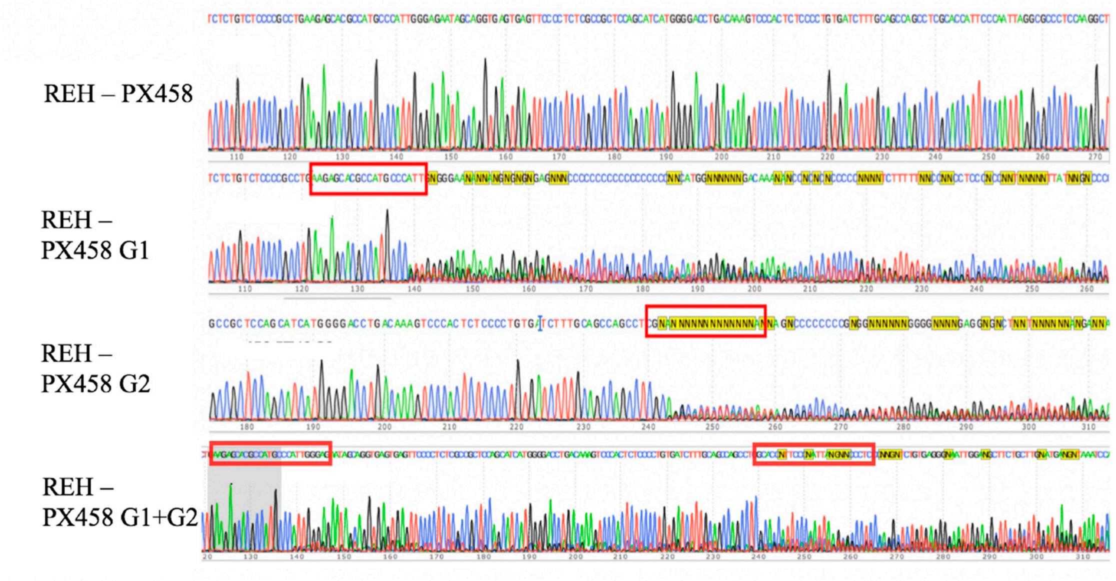

Figure S1. Edited-REH sequences. Sanger sequencing of the *E/R* fusion region in REH cells. The REH cells expressing PX458 without sgRNA, used as a control, had a wild type sequence, while cells expressing G1 sgRNA, G2 sgRNA or G1 + G2 sgRNAs showed a mixture of sequences around the expected Cas9 cleavage point. sgRNA guide sequences are shown in red box.

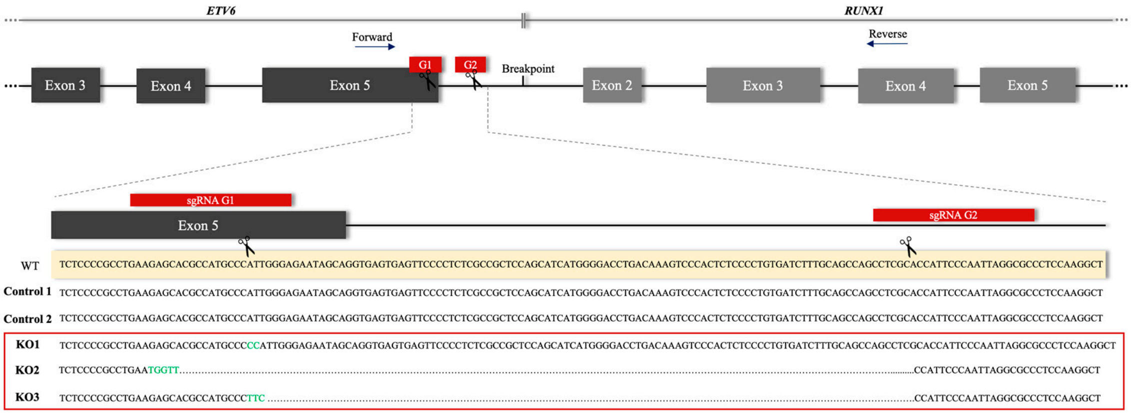

Figure S2. Edited sequences of single cell derived-cell line. The pool of REH edited-cells with both guides (G1 + G2) was separated by single cell. The sequence corresponding to the *E/R* fusion region was studied by Sanger sequencing in all clones. Two clones with single-non-edited sequence and therefore, with a wild type sequence, were selected as control clones. On the other hand, three single-edited cell clones with *E/R* KO sequence were selected as KO clones (KO1, KO2 and KO3). The pair of oligonucleotides used for ETV6/RUNX1 RT-qPCR is showed with blue arrows. These oligonucleotides were designed outside the editing region (exon 5 of *ETV6,* sense, 5 – CTCTGTCTCCCCGCCTGAA – 3 and exon 4 of

RUNX1, antisense, 5 – CGGCTCGTGCTGGCAT -3) and with a distance between them in mRNA of 143 base pairs (bps)

A

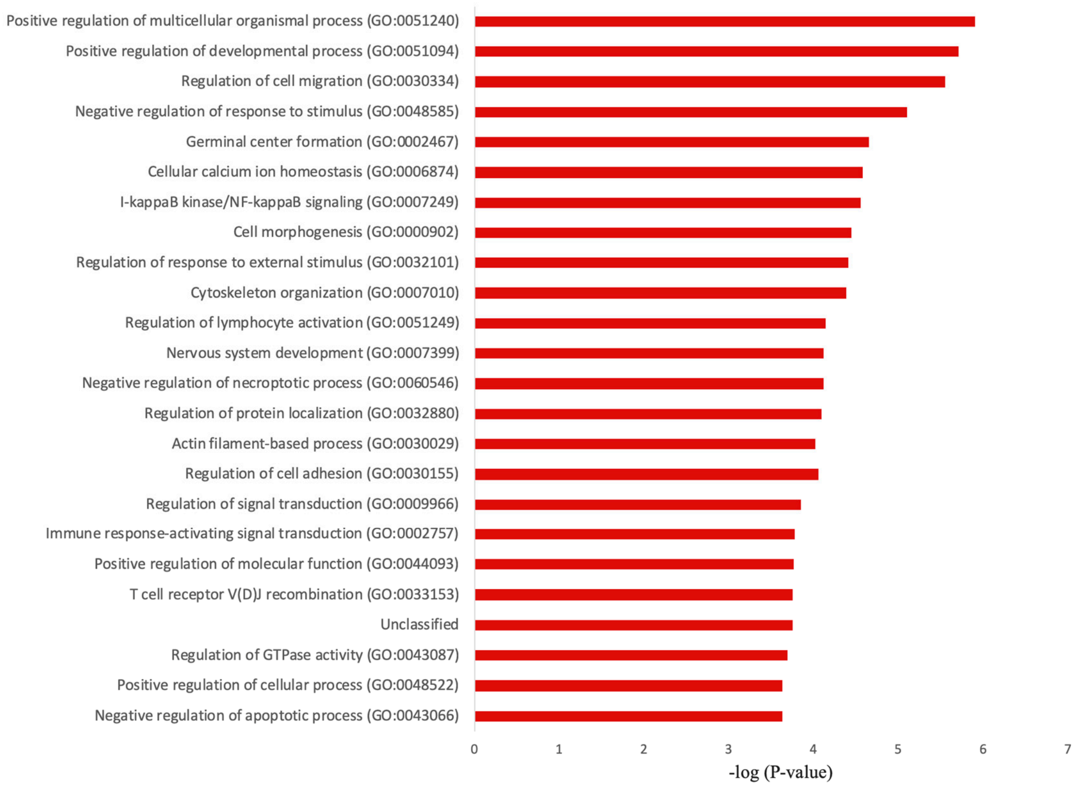

B

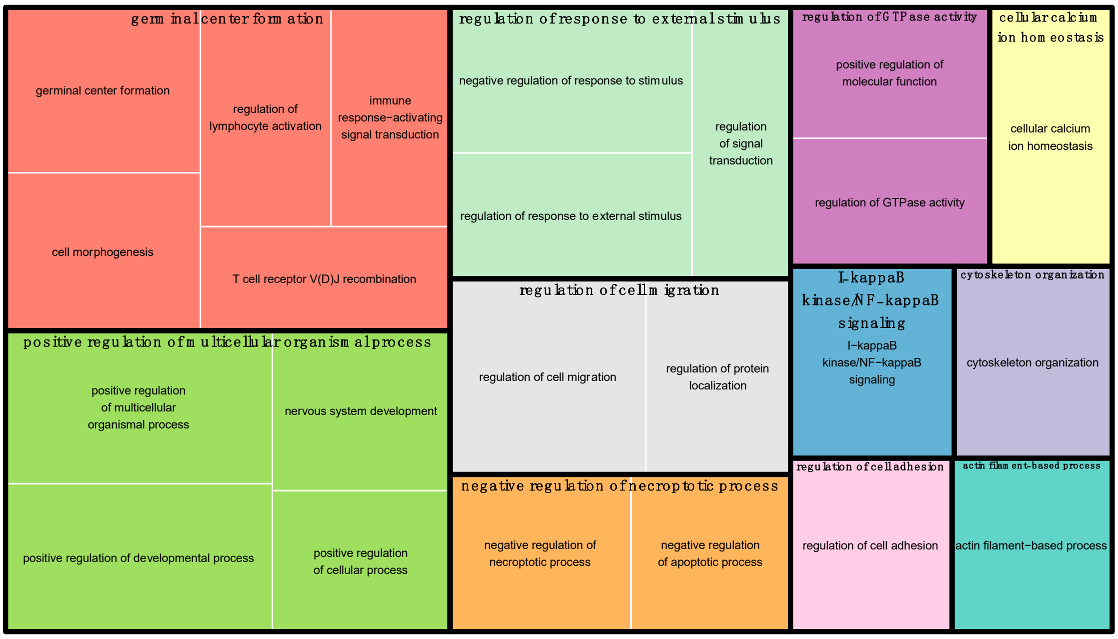

Figure S3. GO Enrichment Analysis. (A) This figure shows the biological processes obtained through enrichment analysis according to -log(P-value). (B) Each rectangle is a single cluster representative. The representatives are joined into 'superclusters' of loosely related terms, visualized with different colors. Size of the rectangles varies according to the P-value.

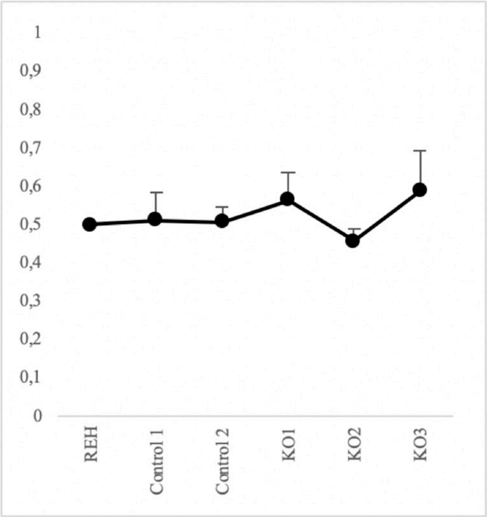

Figure S4. MTT proliferation assay. No proliferation changes were observed in E/R KO clones respected to REH cells and control clones at 72 hours by MTT assay. This experiment had 3 replicates.

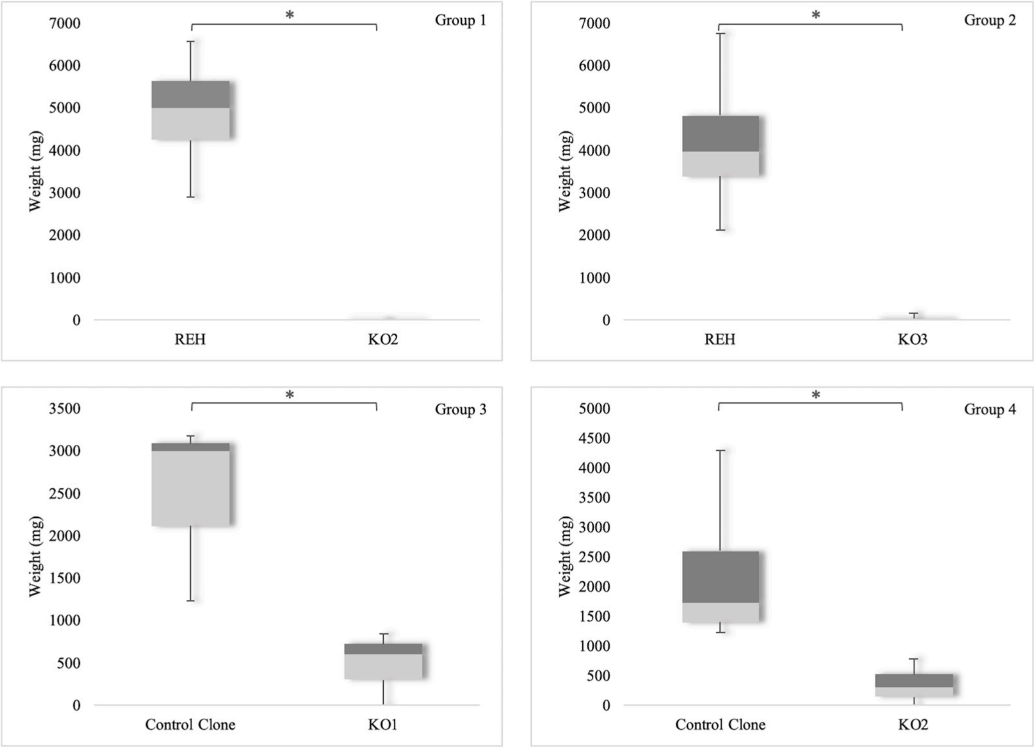

Figure S5. Measure of tumor growth. Mean of the tumor weight for each group of mice, represented by box plot, after being sacrificed at day 48 (group 1 and 2) or day 62 day (group 3 and 4). In this image, the mean of the sizes of the tumors is represented by box plot. In each group they are compared according to

the cells injected in those mice. All the mice belonging to the same group were sacrificed on the same day. Each group had 4 replicates. *P<0.05 (unpaired t-test).

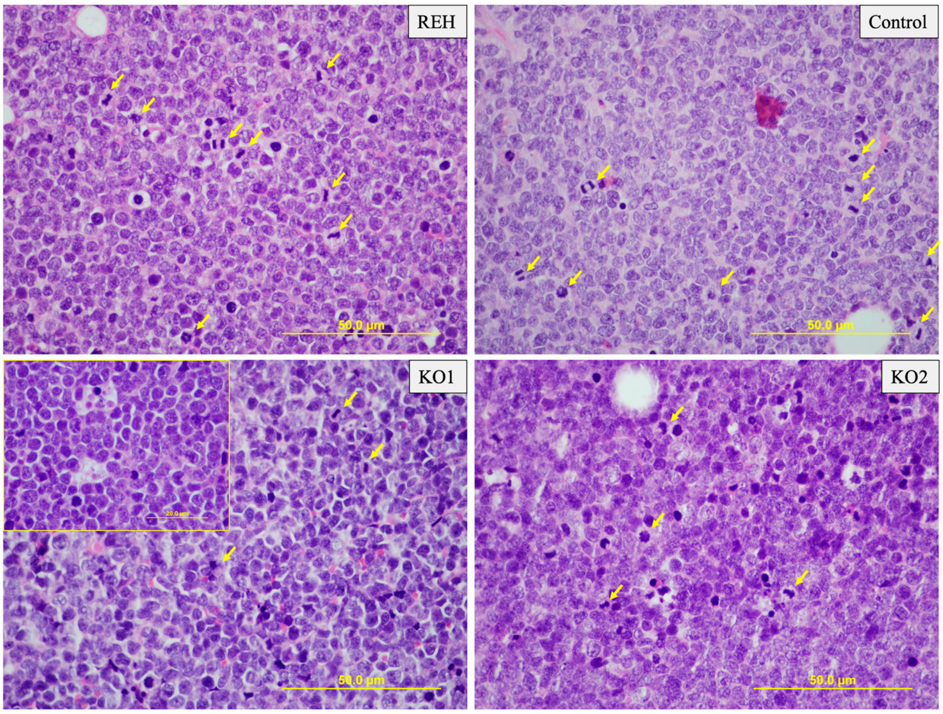

Figure S6. Tumor growth and histopathological findings. H&E representative areas of tumors from each group. Tumors were composed of monomorphic cells with round nucleus. Although mitotic figures (yellow arrows) are observed in all tumors, a higher number were observed in REH and control tumors. Macrophages are shown in KO1 clone (box in the upper left corner).
